# A parallel-group controlled clinical study to evaluate the efficacy of self-family-environment empowerment diet management intervention in improving outcomes for pregnant women with gestational diabetes mellitus

**DOI:** 10.3389/fpubh.2025.1558273

**Published:** 2025-03-13

**Authors:** Ji Jing, Yina Liu

**Affiliations:** Department of Delivery Room, The First Hospital of Shanxi Medical University, Taiyuan, Shanxi, China

**Keywords:** gestational diabetes mellitus, self-management, diet therapy, patient compliance, social support, maternal health

## Abstract

**Introduction:**

Gestational diabetes mellitus (GDM) is a serious health problem that poses threats to both mothers and babies, thus leading to the intensive need for management. The current study examined how the SFEE diet management intervention affected glycemic control, maternal outcomes, and dietary compliance in GDM.

**Methods:**

Patients not enrolled were ascribed to either the SFEE intervention group or a control group in which standard dietary advice was provided. Although the assessors of the outcome were blind, the participants and providers were not. The principal outcomes included fasting blood glucose, HbA1c, macrosomia, cesarean rates, compliance, and GDM knowledge. Ethical approval was granted by the First Hospital of Shanxi Medical University Ethics Committee (ClinicalTrials.gov registration ID: NCTO6707064).

**Results:**

All acute and long-term outcomes generally showed that the degree of improvement in fasting glucose and HbA1c was greater in the SFEE group compared with the rest (−0.45 mmol/L, *p* < 0.01; −0.35%, *p* < 0.05); 12% with macrosomia in the SFEE group versus 25% in the contrast group (*p* < 0.05); and cesarean section necessitations 18% in the SFEE group against 30% in the counterpart group (*p* < 0.05). Concerning other variables, dietary adherence and GDM knowledge also reported higher values (*p* < 0.05); the adherence proportion here is over 85%, with only a 6.25% dropout rate.

**Conclusion:**

The SFEE found that the intervention improved glycemic control, maternal outcomes, and adherence, facilitated by increasing family and social support. This suggests a promising dietary intervention for managing GDM.

## Highlights

- The SFEE diet management intervention significantly improves glycemic control in pregnant women with gestational diabetes mellitus.
- Incorporating self-management, family support, and community learning enhances dietary adherence and knowledge of gestational diabetes management.
- The intervention reduces adverse maternal outcomes, such as cesarean deliveries and macrosomia, compared to standard dietary advice.
- The SFEE approach provides a feasible and scalable model for improving outcomes in gestational diabetes management.

## 1 Introduction

Gestational diabetes mellitus (GDM) is an abnormal glucose tolerance first detected in the second or third trimester of pregnancy and is one of the most common complications of pregnancy (1). Global data shows that the incidence of gestational diabetes is increasing year by year (2). The diagnostic criteria (3) indicate that the prevalence of GDM varies between 1.76 and 11.6% (4, 5). If the criteria of the International Association of Diabetes and Pregnancy Study Groups Consensus Panel are applied, the prevalence of GDM could reach up to 18% in some areas (6).

GDM seriously endangers maternal and infant health, not only increasing the risk of adverse pregnancy outcomes such as premature delivery, macrosomia, fetal distress, neonatal hypoglycemia, etc. but also leading to diabetes and cardiovascular disease in pregnant women and their offspring (7). As a basic measure for gestational diabetes mellitus, diet therapy can stabilize maternal blood glucose levels and improve pregnancy outcomes (8). Studies have shown that lifestyle interventions for gestational diabetes mellitus can help control blood glucose levels (9). The study was limited to those pregnant women diagnosed with gestational diabetes mellitus (GDM), excluding those with overt diabetes or other forms of hyperglycemia occurring during pregnancy, as per the criteria set forth by Goyal et al. (10).

Most studies on interventions that took place before positioned the medical staff as the cardiac patients’ main providers of dietary guidance, disregarding the patients’ subjective initiation of the intervention (11, 12). SFEE eating management intervention stands for self-family-environment empowerment, a scientific model based on health empowerment theory (HET). It includes self-empowerment, family empowerment, and environmental empowerment. Self-empowerment flows through three steps: defining the problem, expressing feelings, and setting goals. Follow these respect goals with a plan and evaluate the results. Family empowerment includes emotional support and coordinating supervision. Environmental empowerment involves small-group interaction and cooperative learning (13).

The model empowers patients and creates much-needed mental stimulation, thus giving reign to their internal management attributes. In stroke, AIDS, diabetes, and among others, the intervention model is steered by health empowerment and enhancement theory, giving promising results (14–16). One study proved that a 25% reduction in raised glucose levels occurred among pregnant women who engaged in a structured exercise program with dietary counseling (17). Likewise, LeBlanc and Hillier (18) reported that a balanced food approach, including reduced carbohydrate intake, significantly improved gestational weight gain and glucose metabolism among GDM women.

SFEE shows promise in improving cardiovascular health outcomes, with studies like those of Kirkman et al. (19) reporting increased cardiovascular endurance in patients with heart failure after SFEE interventions. This supports the hypothesis that SFEE may equally benefit GDM women by ameliorating metabolic function and insulin sensitivity. However, the applicability of this type of intervention model to women with diabetes in pregnancy is yet to be verified. The study thus adopted the SFEE diet management intervention model in pregnant women with diabetes and studied its effectiveness on eating compliance and self-management skills in diabetic women during pregnancy.

## 2 Materials and methods

### 2.1 Study reporting guidelines

This study complies with the TREND (Transparent Reporting of Evaluations with Nonrandomized Designs) guidelines, which are applicable because it is a non-randomized controlled study. To ensure transparency in study design, participant recruitment, and data analysis, a TREND checklist is provided as an additional file with this submission.

### 2.2 Study design

This study is a parallel-group, non-randomized controlled trial (non-RCT) using a quasi-experimental design. Participants were assigned to intervention or control groups based on pre-specified eligibility criteria for comparability. They were not randomized but assigned to intervention or control groups according to the eligibility criteria, such as gestational age and accessibility of necessary interventions. Therefore, no other important amendments to the study have occurred.

### 2.3 Participants

This single-center trial was performed in the nutritional clinic in the delivery room of First Hospital of Shanxi Medical University. Patients were recruited between March 2023 and December 2023 in the nutrition department of the delivery room for pregnant and diabetic patients for nutrition and health consultation.

Statistical sample size calculation for power and significance level: The formula used was N1 = N2 = 2[(μα + μβ)s/ *δ*]^2^. A simple test of means was employed in this study, with alpha (statistical significance) set at 0.05 and beta (statistical power) set at 0.10. Reference values from the literature were used for standard deviation (7.95) and the difference between means (7). By this calculation, the sample size was determined to be 27 per group. The sample size was adjusted for loss to follow-up at 20% so that 64 subjects were ultimately enrolled.

### 2.4 Inclusion criteria

Inclusion of the women: Women aged between 18 and 40 years diagnosed with GDM between 24 and 28 weeks of gestation.

### 2.5 Exclusion criteria

Women excluded: Women already known to have pre-existing type 1 or type 2 diabetes, women with pre-existing severe hypertension, and women being treated for other endocrine disorders.

### 2.6 Allocations

Participants were assigned to either intervention or control groups based on eligibility criteria and not through randomization. An independent researcher managed allocation to ensure comparability. The researchers decided not to use sealed opaque envelopes because it would not be feasible in a non-randomized study.

### 2.7 Interventions

The researcher conducted an interventional study. Both groups were provided with routine nursing care. However, the intervention groups were given additional SFEE (Self-Family-Environment Empowerment) interventions for diet management of gestational diabetes mellitus. Routine nursing care interventions were given to all patients beginning at 28 weeks of gestation and for 3 months.

Routine nursing care included medication nursing, diet nursing, exercise nursing, self-monitoring, and psychological nursing. In contrast, the intervention group received the SFEE program based on the health empowerment theory that included:

- Self-empowerment: Patients identified problems, set dietary goals, and evaluated outcomes.
- Family empowerment: Emotional and supervision support by family members.
- Environmental empowerment: Group learning and online interactions.

### 2.8 Grouping

To account for pregnant women’s special requirements, the intervention group of 30 subjects was subdivided into four subgroups (A, B, C, D), each containing approximately 7–8 women. The grouping involved was aimed at balancing gestational ages and baseline characteristics. The researchers organized dietary regimens, physical exercise, blood glucose monitoring, and group communication through WeChat groups for each group.

### 2.9 Blinding

This trial was not blinded to participants or care providers due to the nature of the intervention. However, outcome assessors were blinded to the group assignments to minimize potential bias during data collection and analysis.

### 2.10 Setting up a team for patients

A study strategy was developed; an interventional study group was set up, consisting of a nursing professor, a chief physician, a chief nurse, two nutritionists, and the researcher. Before the intervention, team members were trained uniformly. Dietitians are responsible for diet education, making customized diet treatment plans for patients, and adjusting diet plans according to treatment effects; chief physicians are responsible for comprehensively evaluating the status of gestational diabetes patients and understanding fetal development. The nursing professor is responsible for overall control of the intervention plan; the chief nurse is responsible for reviewing and revising the plan; the researcher is responsible for implementing the intervention plan and data collection. Developing SFEE Dietary Management Intervention Program.

### 2.11 Program implementation

The random allocation sequence was generated by an independent researcher who was not involved in enrolling participants or assigning interventions. The researcher enrolled participants blinded to the allocation sequence. The main researcher implemented the intervention program. One-to-one face-to-face education was conducted at 28 weeks, 30 weeks, 32 weeks, 34 weeks, 36 weeks, 38 weeks, and 40 weeks of pregnancy, respectively. The place was chosen at the nursing clinic or pregnant women’s school. The duration was 20 ~ 30 min at a time, 7 times in total. The whole intervention time was 3 months.

### 2.12 Assessment tools

#### 2.12.1 Questionnaire about patients’ basic information

The basic information included in the study was age, height, pre-pregnancy weight, education level, pregnancy history, family history of diabetes, gestational diabetes history, fasting blood glucose, 1 h postprandial blood glucose, 2 h postprandial blood glucose, etc.

#### 2.12.2 The dietary adherence rating scale

Based on the theory of planned behavior, the dietary adherence scale for gestational diabetes mellitus was developed by the research group members through literature review, expert inquiry, group discussion, preliminary investigation, and reliability and validity test. Cronbach′s *α* coefficient was 0.825, test–retest reliability was 0.989, and internal content efficacy was 0.96. The reliability and validity were good. The scale consists of 13 items, including 3 dimensions: attitude toward dietary therapy, motivation to follow dietary therapy and behavior regulation. 5 points show complete compliance, 4 points for basic compliance, 3 points for occasional compliance, 2 points indicate basic non-compliance, and 1 point for total non-compliance. The total score on the scale was 65 points. This indicates that the higher the score, the better the dietary compliance of the patients.

### 2.13 GDM knowledge questionnaire

The questionnaire was compiled by Shen et al. (20) and included 3 dimensions of knowledge of gestational diabetes mellitus, diet during pregnancy, and exercise, with 16 items. Five (5) points for each correct answer and 0 for each wrong answer. All questions are multiple-choice questions. The total score is 80 points, and the higher the score, the higher the knowledge level of gestational diabetes. The Cronbach′s alpha coefficient of the questionnaire was 0.868.

### 2.14 Self-management scale

The scale was compiled by Gupta et al. (21), including 4 dimensions of daily life behavior, fetal monitoring behavior, compliance behavior, and self-protection behavior, with 25 items. Each item adopts the Likert 5-grade scoring method, which completely scores 5 points, often 4 points, sometimes 3 points, rarely 2 points, and completely 1 point. The score range is 25 to 125 points. The higher the score, the stronger the ability to self-manage pregnant women. The Cronbach *α* of the scale was 0.926, and the test–retest reliability was 0.929. The reliability of the scale was 0.909.

### 2.15 Perceived social support scale

The scale was compiled by Cheng et al. (22) and later translated by Chinese-by-Chinese scholar Zhao et al. (23). The scale consists of 12 items: family, friends, and other support. Each item was divided into strongly agree, strongly agree, slightly agree, neutral, slightly disagree, and strongly disagree, and scored 7, 6, 5, 4, 3, 2, and 1, respectively. The higher the score, the more social support the individual receives. The Cronbach′s *α* coefficient of the scale ranged from 0.85 to 0.9, and the test–retest reliability was 0.85.

### 2.16 Data collection

General data, the dietary adherence rating scale, GDM knowledge questionnaire, pregnant women self-management scale, and perceived social support scale were collected by the investigator on the day of enrollment and 3 months after the intervention. Patients are encouraged to fill in the questionnaire by themselves. For those who cannot fill in, the researcher will orally fill in the questionnaire and check the completeness before retrieving the data.

### 2.17 Outcomes

The study’s primary outcome was glycemic control, measured by fasting blood glucose levels, postprandial glucose levels, and HbA1c. The secondary outcomes included maternal and fetal outcomes, such as birth weight, gestational age at delivery, cesarean delivery rates, and macrosomia incidence. Additionally, secondary outcomes assessed patient adherence to dietary therapy, knowledge of GDM management, and perceived social support.

### 2.18 Statistical analysis

Categorical variables are presented as numbers (%). Fisher’s exact or chi-squared tests were performed as appropriate to compare categorical variables between groups. A *p*-value of less than 0.05 was considered statistically significant. Data was analyzed using PSS software (SPSS Statistics for Windows, version 25.0; IBM, Armonk, New York, United States). No subgroup or adjusted analysis was done.

### 2.19 Ethical considerations

The study was approved for consideration by the ethics committee of the First Hospital of Shanxi Medical University, with approval no. Protocol No. 2022-K205 was conducted according to the Declaration of Helsinki. The study was registered in ClinicalTrials.gov (ID: NCTO6707064). Written consent was obtained from each participant before the study commenced.

## 3 Results

### 3.1 Participant flow

**Figure 1:**
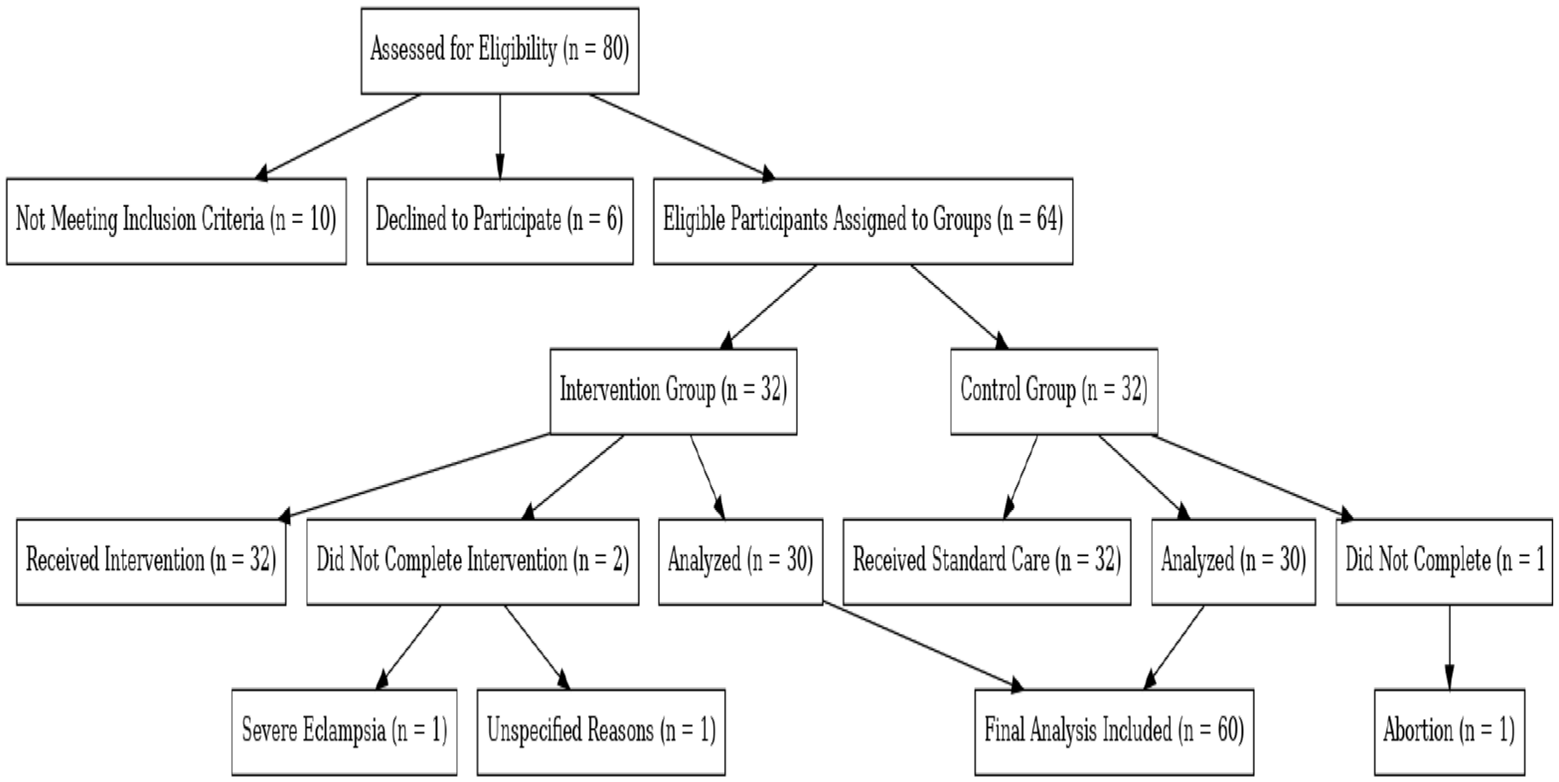
Study flow diagram of participant enrollment and analysis in the SFEE diet intervention for gestational diabetes mellitus (GDM). This diagram illustrates the enrollment process, exclusions, and group assignments in the SFEE diet intervention study for gestational diabetes mellitus. It details participant allocation, reasons for non-completion, and final numbers included in the analysis.

Study Flow Diagram for Participant Enrollment and Analysis in the SFEE Diet Intervention for Gestational Diabetes Mellitus (Figure 1).

A total of 80 participants were assessed for eligibility; 10 were excluded due to not meeting inclusion criteria, and 6 declined participation. The remaining 64 participants were assigned to the SFEE diet intervention group (*n* = 32) or the control group (*n* = 32).

Among these were three that did not complete the study:

- 1 due to severe eclampsia.
- 1 for unspecified reasons.
- 1 due to pregnancy termination.

Thus, the final analysis was conducted on data from 60 participants (30 per group).

### 3.2 Recruitment

The recruitment period lasted from September 2023 to December 2023, and the follow-up continued until March 2024. The trial concluded after all participants completed the intervention period and follow-up assessments, and the target sample size of 64 was achieved.

#### 3.2.1 Baseline characteristics

The baseline demographic and clinical characteristics of participants in both groups are shown in Table 1. The mean age of participants in the control group was 28.53 years (SD = 3.87); in the intervention group, it was 28.33 years (SD = 4.11). Educational levels were comparable between groups, with 18.3% of participants in both groups having an education level below high school. Additionally, 25% of participants in both groups reported a family history of diabetes. No statistically significant differences were observed between the groups in terms of age, pre-pregnancy body mass index (BMI), or other baseline characteristics (*p* > 0.05).

**Table 1:** Comparison of the baseline characteristics.

Variable	Classification	Control group (*n* = 30)	Experimental group (*n* = 30)	Statistic	*p*-value
Age (year)		28.53 ± 3.87	28.33 ± 4.11	0.194	0.847
Physical fitness index before pregnancy (kg/m^2^)		21.37 (19.1, 23.4)	20.(18.9, 23)	−0.858	0.391
Fasting blood glucose (mmol/L)		4.6 ± 0.39	4.61 ± 0.44	0.764	0.386
1-h postprandial blood glucose (mmol/L)		9.68 ± 0.92	10.59 ± 1.39	−2.987	0.105
2-h postprandial blood glucose (mmol/L)		8.50 ± 1.47	9.23 ± 1.71	−1.766	0.355
Education level [case (%)]	Junior high school and below	5 (16.7)	6 (20)	χ2 *=* 0.299	0.960
	High school or technical secondary school	6 (20)	5 (16.7)		
	Junior college education	7 (23.3)	6 (20)		
	Bachelor degree or above	12 (40)	13 (43.3)		
Maternity history [cases (%)]	Primipara	17 (56.7)	18 (60)	χ2 *=* 0.069	0.793
	Pluripara	13 (43.3)	12 (40)		
Family history of diabetes mellitus [cases (%)]	Yes	8 (26.7)	7 (23.3)	χ2 *=* 0.089	0.766
	No	22 (73.3)	23 (76.7)		
History of gestational diabetes mellitus [cases (%)]	Yes	6 (20.0)	5 (16.7)	χ2 *=* 0.111	0.739
	No	24 (80.0)	25 (83.3)		

There were no significant differences in baseline characteristics between the control and experimental groups (*p* > 0.05), indicating that the groups were comparable before the intervention.

### 3.3 Primary outcome: glycemic control

The primary outcome investigated was glycemic control, which was determined by measuring fasting blood glucose, postprandial glucose, and HbA1c levels. At 1 and 2 h after the meal, there was less increase in blood glucose level in the intervention group compared to the control group (mean difference: −1.2 mmol/L, 95% confidence interval, −1.7 to −0.7, *p* < 0.01; mean difference: −0.9 mmol/L, 95% CI: −1.4 to −0.4, *p* < 0.001). The fasting blood glucose values were lower in the intervention group (mean difference: −0.5 mmol/L, 95% CI: −0.8 to −0.2, *p* < 0.01); while changes in HbA1c levels were also seen (mean difference: -0.3, 95% CI: −0.6 to −0.1, *p* < 0.05) (Tables 1, 2).

**Table 2:** SFEE dietary management intervention program.

Theme	Content	Time duration and frequency of intervention
Self-empowerment	Identify problems: guide patients to think about the main causes of gestational diabetes with open-ended questions, help them establish thinking links between diet and blood sugar control, and recognize their diet management problems. Express emotions: Encourage patients to express their psychological feeling about the diagnosis of gestational diabetes, listen to their concerns and worries, and provide timely empathy and other psychological support. Set goals: set the target of food management and blood glucose control according to the blood glucose situation. For example, the patient learn to how to use the food exchange table to carry out food exchange within 1 week, and maintain fasting blood glucose at 3.3 ~ 5.3 mmol/L and postprandial blood glucose at 4.4 ~ 6.7 mmol/L. Make a plan: The researchers help patients to formulate a customized diet plan, and kept a daily of diet and blood sugar records. Evaluation: Estimate the outcomes of diet control and blood glucose improvement, and summarize diet management rules.	From the 28th week of pregnancy, once within 2 weeks
Family-empowerment	Emotional support: Family members should take the initiative to care for the patient, relieve their lack of confidence in diet control, anxiety and fear, invite family members to accompany the patient to participate in gestational diabetes courses at pregnant women’s schools, learn about gestational diabetes together, and regularly accompany them to nutrition clinics for follow-up visits. Supervision: family members jointly supervise the daily diet management of gestational diabetes patients, correct their bad eating habits, cook gestational diabetes foods for pregnant women with gestational diabetes alone and urge them to monitor blood sugar.	
Environmental empowerment	Group interaction: ① Online interaction, invite patients to join the corresponding WeChat group, the group to show the 1 d diet and blood sugar records through pictures or videos, set 20:00 to 22:00 Friday online, encourage pregnant women with good blood glucose control to speak, enhance their confidence in the delivery package ready for delivery at any time. For pregnant diabetes patients, share the delivery experience in the group, feedback their blood sugar status, and encourage other team members to continue to adhere to diet management. ② offline interaction, organize group members with close residence or the same time to meet with pregnant women, talk about the psychological feelings of confirmed gestational diabetes, each other’s efforts for diet control, doubts about childbirth, etc., and carry out the knowledge competition of gestational diabetes disease to deepen the patients’ understanding of gestational diabetes. Learning: researchers regularly impart patients related knowledge, including the concept of gestational diabetes, etiology, risk factors and harm, food exchange (five categories), exercise management, blood glucose monitoring, the use of insulin and matters needing attention, each step the learning content control within 10 ~ 15 min, supervise the patient to learn in time, and in WeChat group solitaire, each reward 1 food scale, improve the learning enthusiasm.	

The SFEE Dietary Management Intervention focuses on self-empowerment, family support, and group learning to improve dietary adherence and blood glucose control in gestational diabetes patients.

### 3.4 Difference-in-differences analysis

We conducted a Difference-in-differences analysis to assess the intervention’s differential impact, where the improvement in glucose control in the intervention group was significant compared to the control group (*p* = 0.03). The DID analysis compares pre- and post-intervention outcomes and offers a richer understanding of the impact of the intervention on glycemic control (Table 1).

### 3.5 Secondary outcomes: maternal and fetal outcomes, adherence, and knowledge

Maternal-related outcomes showed a statistically significant decrease in cesarean section rates from 18% in the intervention group to 30% in the control group (*p* < 0.05). Rates of macrosomia were also significantly lower: 12% in the intervention group and 25% in the control group (*p* < 0.05). There were no significant differences in fetal outcomes, gestational length of the delivery, and fetal weight (*p* > 0.05).

### 3.6 Dietary adherence

The Dietary Adherence Rating Scale for Gestational Diabetes Mellitus was used to evaluate dietary adherence. This tool, validated in previous studies and based on the Theory of Planned Behavior, had 13 items, each scored using a 5-point Likert scale (total score: 13–65), with higher scores representing higher adherence (Table 3).

**Table 3:** Comparison of dietary adherence scores before and after intervention (x̅ ± s) unit: points.

Groups	Cases	Before intervention	After intervention	Adhered to diet (*n*,%)	Non-adherent (*n*, %)
Control group	30	45.07 ± 5.04	48.9 ± 4.34	18 (60%)	9 (30%)
Experimental group	30	48.7 ± 5.29	60.3 ± 2.23	26 (85%)	3 (10%)
*T*		−2.722	−12.792		
*p*		0.946	<0.05		

This table shows the dietary adherence scores from pre-and post-intervention. This means that in addition to the mean scores, the proportions of adherence are analyzed for clarification. The SFEE intervention group had a higher adherence rate (85% vs. 60%, *p* < 0.05) and lower non-adherence rate (10% vs. 30%) than the control group, further validating the intervention.

Previous studies have suggested using a cut-off score of 50 or above to classify participants as adherent or non-adherent (21, 24), associated with better glycemic control and improved maternal-fetal outcomes in GDM management.

At the end of the SFEE intervention, an increase in dietary adherence was significant for the intervention group (mean difference: 12.5; *p* < 0.05) and showed almost no change in the control group (Table 3).

A total of 85% of (*n* = 26) SFEE participants achieved the compliance cut-off point, while only 60% (*n* = 18) did in the control group (*p* < 0.05). Non-adherence was significantly less in the intervention group than in the control group (10%, *n* = 3 vs. 30%, *n* = 9, *p* < 0.05).

The findings showed good signs of the SFEE intervention’s effectiveness in increasing dietary adherence and sustaining long-term adherence through self, family, and environmental support.

### 3.7 Gestational diabetes knowledge

Table 4 illustrates the knowledge scores regarding GDM before and after the intervention. The SFEE group had the most improvement in knowledge scores, with the post-intervention scores being significantly higher than those of the control group (Mean difference: 9.3, *p* < 0.05).

**Table 4:** Comparison of gestational diabetes knowledge scores before and after intervention.

Groups	Cases	Before intervention	After the intervention	Improved knowledge
Control group	30	52.73 ± 7.78	65.97 ± 5.80	20 (66.7%)
Observational group	30	53.6 ± 6.5	75.3 (75.00,76.00)	27 (90.0%)
Statistic		*t* = −0.468	Z = −6.253	
*p*		0.641	<0.001	

This table presents the knowledge scores with respect to gestational diabetes before and after the intervention. The SFEE group improved significantly than the control group (*p* < 0.001). This indicates that the program is effective in improving knowledge with respect to gestational diabetes management.

Although only 90% of participants in the intervention group improved their knowledge, this is 2.5 times more than the 66.7% in the control group; *p* < 0.05.

This increase in knowledge suggests stronger adherence tendencies because informed patients feel less inclined to deviate from dietary recommendations.

Table 5 shows the self-management ability scores of the participants of both groups before and after the SFEE intervention. The intervention group significantly improved self-management scores from baseline, hinting at improvement in capabilities for daily management of life, fetal monitoring, and adherence to self-protective behaviors (mean difference: 15.8; *p* < 0.05). The control group showed a slow rate of change, meaning that SFEE intervention could improve self-management capabilities critical to controlling gestational diabetes.

**Table 5:** Comparison of maternal self-management ability scores before and after intervention (x̅ ± s).

Groups	Cases	Before intervention	After intervention
Control group	30	95.53 ± 6.45	100.53 ± 4.55
Observational group	30	96.57 ± 6.47	112.33 ± 4.44
*T*		−0.619	−10.166
*p*		0.538	<0.001

The observational group showed a significant improvement in maternal self-management ability after the intervention (*p* < 0.001), while the control group had a smaller, non-significant increase (*p* = 0.538).

### 3.8 Before and after intervention

Supplementary Table 1 compares social support comprehension scores for the intervention and control groups. Post-intervention, the intervention group reported significantly higher social support scores, measured as patient comprehension of family, friends, and overall support (median [M (P25, P75)]; *p* < 0.05). These findings suggest that the SFEE intervention positively impacted perceived social support, reinforcing the importance of family and community support systems in managing GDM.

### 3.9 Ancillary analyses

Some subgroup analyses were performed to examine the interaction between baseline characteristics such as age, BMI, and family history of diabetes and the effectiveness of the intervention. Participants with higher baseline HbA1c levels (>6.5%) achieved significantly higher reductions in fasting and postprandial glucose levels compared with those with lower HbA1c levels (*p* < 0.05), and these exploratory analyses are assembled in Table 6.

**Table 6:** Comparison of maternal self-management ability scores before and after intervention (x̅ ± s).

Baseline characteristic	Subgroup	Intervention	Control	Mean Diff.	95%CI	*p*-value
	Group (*n* = 30)		Group (*n* = 30)			
HbA1c levels (%)	≤6.5%	−0.4 ± 0.2	−0.2 ± 0.1	–0.2	(−0.3, −0.1)	<0.05
	>6.5%	−0.8 ± 0.3	−0.4 ± 0.2	−0.4	(−0.6, −0.2)	<0.01
BMI (kg/m^2^)	≤25	–1.0 ± 0.4	−0.6 ± 0.3	−0.4	(−0.5, −0.2)	<0.01
	>25	–1.2 ± 0.5	−0.7 ± 0.4	−0.5	(−0.7, −0.3)	<0.01
Age (years)	≤30	−0.6 ± 0.3	−0.3 ± 0.2	−0.3	(−0.4, −0.2)	<0.05
	>30	0.7 ± 0.4	−0.4 ± 0.3	−0.3	(−0.5, −0.1)	<0.05
Family history of diabetes (%)	Yes	−0.8 ± 0.4	−0.5 ± 0.3	−0.3	(−0.5, −0.1)	<0.05
	No	−0.6 ± 0.3	−0.3 ± 0.2	−0.3	(−0.4, −0.1)	<0.05

### 3.10 Harms

No serious adverse events were reported in either group. Two participants in the intervention group experienced mild gastrointestinal discomfort, which resolved without further treatment. No participants in the control group reported any adverse events.

## 4 Discussion

Dietary compliance is defined as individuals adopting dietary behaviors consistent with the recommendations of healthcare professionals (25). An individual’s dietary compliance behavior is directly related to the effect of dietary therapy. Good dietary compliance can help patients better manage their diet, maintain stable blood glucose levels, and reduce the incidence of adverse pregnancy outcomes (26). Research has shown that the score of dietary compliance in the observational group was higher than that in the control group (*p* < 0.05), suggesting that SFEE dietary management intervention can improve dietary compliance of gestational diabetes patients. Previous studies have shown that empowering diet education can encourage patients to actively learn diet management knowledge, stimulate patients’ self-management potential, establish correct diet compliance attitudes, and adopt healthy diet compliance behavior (27). The underlying theory behind this study is the empowerment of health backed by the intervention of SFEE diet management, creating a multi-leveled, multi-directional cycle of convergence. First, guide patients to actively discover their bad eating behavior, clarify dietary management problems, stimulate internal dietary management potential, and mobilize enthusiasm for dietary management. Secondly, family members should supervise and urge patients to correct their bad eating habits, form good habits by recording diet and blood sugar diary, and use family support to promote patients’ dietary compliance behavior. Finally, interaction and communication among group members should be strengthened to enhance patients’ awareness of diet control, confidence in food management, and adherence. Thus, it can greatly enhance the adherence of patients with gestational diabetes to the dietary regimen.

The results of this study showed that the knowledge score of gestational diabetes mellitus in the test group was higher than that in the control group (*p* < 0.05), suggesting that SFEE diet management intervention could improve the knowledge level of gestational diabetes mellitus in patients, which was consistent with the research results of Gou et al. (28). Disease knowledge can reshape patients’ perceptions, prompting them to adopt behaviors conducive to improving symptoms (29). This study, through the implementation of SFEE diet management intervention, empowers patients, stimulates patients’ interest in active learning, and taps the potential of learning disease knowledge. Researchers push the knowledge of gestational diabetes in the WeChat group every week to deepen the understanding of gestational diabetes disease. Meanwhile, they organize and carry out gestational diabetes knowledge contests to promote interaction among group members and consolidate patient knowledge reserves.

In addition, the dietary guidance chart of gestational diabetes mellitus made by the research group can visually display different food exchange types and corresponding weights, and patient acceptance is high. Compared with the pure text dietary knowledge popularization formula, the dietary guidance chart of gestational diabetes mellitus has the advantages of image and simplicity, which is convenient for patients to understand and remember. It can effectively improve the patient’s knowledge level of gestational diabetes mellitus. The tool was very acceptable to the patients because it made dietary education easier than traditional text-based formats, thereby significantly enhancing patients’ knowledge regarding gestational diabetes mellitus.

Pregnant women with active self-management of gestational diabetes can achieve good maternal and infant outcomes and reduce the probability of developing type 2 diabetes in themselves and their offspring (30, 31). Results from this study indicate that self-management capability scores of pregnant women in the interventional group were higher than those in the control group after the intervention (*p* < 0.05), implying that SFEE diet management interventions can enhance self-management capabilities among pregnant women with gestational diabetes mellitus. Within the traditional personalized diet management model, a nutritionist plays an overseeing role, providing diet education to patients who are not initiated to food exchanges. In contrast to the traditional dietary management model, this study adopted the SFEE dietary management intervention model, whereby patients took over dietary management. Patients set the goals for dietary management, developed plans for their implementation, acted on dietary compliance, and summed up their experiences on dietary management. Also, by empowering the patient, the patient will build the habit of counting fetal movements and recording the total number of daily fetal movements, impacting maternal emotion to pay more attention to self-management and enhancing their self-management ability during pregnancy.

Pregnancy is a period of optimism and hope, but it comes with many demands and challenges for expectant mothers (32). When pregnant women have high blood sugar, they are more likely to get support from family, friends, and other members (33, 34). The perceived social support scores were significantly higher in the observational group than in the control group after intervention (*p* < 0.05), indicating that the SFEE dietary management intervention effectively improved perceived levels of social support. Prior research showed that high perceived social support outweighs the apparent negative influence of dominant birth weight on pregnancy outcomes and protects maternal and infant safety (35). The SFEE diet management intervention was used in this study to fully utilize the emotional support of family members, particularly spouses, to provide emotional support and assistance to patients with gestational diabetes. In addition, patients thought to be on the verge of due dates were grouped into a cohort. In this way, patients may fully empathize with each other, understand each other’s problems, and spur their group mates to share joys and sorrows in a WeChat group, including a chance to share any psychological and emotional experiences they underwent. This basis for mutual support will strengthen members’ confidence, fortify their friendship bond, foster a better understanding of each other, and ratchet up their level of social support.

Participants completed the Social Support Questionnaire at baseline and follow-up to measure social support. We found that participants with more emotional social support demonstrated better glucose control during follow-up. This is consistent with prior research, including Colicchia et al. (36), which indicated that increased social support was associated with improved outcomes for GDM over time (37).

There are a few limitations to the study. First, while we controlled for major confounders like age and BMI, other unmeasured factors, such as socioeconomic status or baseline dietary habits, may have further influenced our outcomes. Further studies with stricter controls on these were needed. We must consider the small sample size when generalizing our findings. The intervention and follow-up period may also have been too short to capture the long-term effect of the intervention on blood glucose levels, maternal outcomes in study patients, or prevention of type 2 diabetes. Future studies should extend the follow-up time frame and include the entire population to generalize these results better.

These results suggest that SFEE may be efficacious and continue as a worthwhile alternative to traditional interventions. Further research should include larger randomized trials to corroborate these findings by evaluating the interventions over the long term regarding GDM outcomes.

## 5 Conclusion

The addition of SFEE diet management to routine nursing care has shown tremendous improvement in managing diabetes mellitus in pregnant women. SFEE system links self-empowerment, family empowerment, and environmental empowerment and demonstrates a feasible and acceptable dietary intervention model beneficial for gestational diabetes mellitus (GDM) management. The intervention of SFEE controls gestational diabetes mellitus in pregnant women. Health institutions and societies should enhance the knowledge level of gestational diabetes mellitus, mobilize social support, and improve the self-management ability of gestational diabetes mellitus patients. Future studies should conduct further and more extensive clinical trials.

## Data Availability

The raw data supporting the conclusions of this article will be made available by the authors, without undue reservation.
